# Combination therapy with oncolytic viruses and immune checkpoint inhibitors in head and neck squamous cell carcinomas: an approach of complementary advantages

**DOI:** 10.1186/s12935-022-02846-x

**Published:** 2023-01-05

**Authors:** Hui Dong, Mengli Li, Chen Yang, Wei Wei, Xianglei He, Gang Cheng, Shibing Wang

**Affiliations:** 1 Department of Stomatology, Bengbu Medical College, 2600 Donghai Avenue, Bengbu, 233030 China; 2 Department of Stomatology, Center for Plastic and Reconstructive Surgery, Cancer Center, Zhejiang Provincial People’s Hospital (Affiliated People’s Hospital, Hangzhou Medical College), Hangzhou, 310014 Zhejiang China; 3 Department of Ultrasound Medicine, Cancer Center, Zhejiang Provincial People’s Hospital (Affiliated People’s Hospital, Hangzhou Medical College), Hangzhou, 310014 Zhejiang China; 4 Postgraduate Training Base of Jinzhou Medical University (Zhejiang Provincial People’s Hospital, Affiliated People’s Hospital, Hangzhou Medical College), Hangzhou, 310014 Zhejiang People’s Republic of China; 5 Department of Pathology, Cancer Center, Zhejiang Provincial People’s Hospital (Affiliated People’s Hospital, Hangzhou Medical College), Hangzhou, 310014 Zhejiang China; 6 Cancer Center, Key Laboratory of Tumor Molecular Diagnosis and Individualized Medicine of Zhejiang Province, Zhejiang Provincial People’s Hospital (Affiliated People’s Hospital, Hangzhou Medical College), Hangzhou, 310014 Zhejiang China

**Keywords:** Oncolytic viruses, Immune checkpoint inhibitor, Head and neck squamous cell carcinomas, Combination therapy

## Abstract

Squamous cell carcinomas are the most common head and neck malignancies. Significant progress has been made in standard therapeutic methods combining surgery, radiation, and chemotherapy. Nevertheless, the 5-year survival rate remains at 40–50%. Immune checkpoint inhibitors (ICIs) are a new strategy for treating head and neck squamous cell carcinomas (HNSCCs). Still, the overall response and effective rates are poor, as HNSCCs are ‘cold’ tumors with an immunosuppressive tumor microenvironment (TME), limiting ICI’s beneficial effects. In this case, transforming the tumor suppression microenvironment before using ICIs could be helpful. Oncolytic viruses (OVs) can transform cold tumors into hot tumors, improving the situation. Talimogene laherparepvec (T-VEC), oncolytic immunotherapy authorized for advanced melanoma, also showed good safety and antitumor activity in treating head and neck cancer and pancreatic cancer. In combination with pembrolizumab, T-Vec may have more anticancer efficacy than either drug alone. Therefore, understanding the mechanisms underpinning OVs and their potential synergism with ICIs could benefit patients with HNSCC.

## Introduction

Head and neck cancers (HNCs) include lip, oral cavity, larynx, nasopharynx, oropharynx, and hypopharynx malignancies. In 2020, HNCs accounted for 4.7% of all cancers (878,348 new cases) and 2.7% of all cancer deaths (444,347 deaths) worldwide [1]. Head and neck squamous cell carcinoma (HNSCC), originating from the mucosal epithelium, is the most prevalent head and neck malignancy. Its etiology is generally correlated with exposure to tobacco carcinogens, excessive drinking, or both [2, 3]. Increasingly, oropharyngeal tumors are associated with human papillomavirus (HPV) infections, mainly HPV-16 and, to a lesser extent, HPV-18, HPV-52, and other strains [4, 5]. Besides the above risk factors, epidemiological studies have found that, where areca has a significant consumption rate, such as in some provinces of China, there is a high incidence rate of oral squamous cell carcinoma [6]. Despite massive improvements to therapies, such as surgery, radiotherapy, and chemotherapy, the rate of 5-year overall survival (OS) of HNSCC remains low. Up to 25% of patients develop second cancer within five years after diagnosis [7].

Immune checkpoint inhibitors (ICIs) are hot research topics in the clinical treatment of HNSCC [8]. ICIs are more effective against tumors with high tumor-infiltrating lymphocytes (TILs), referred to as ‘hot’ tumors in immunology [9]. However, the levels of TILs in the HNSCC tumor microenvironment are minimal, HNSCC belongs to ‘cold’ tumors [10], and the clinical efficacy of ICIs is abysmal. Oncolytic viruses (OVs), genetically engineered or naturally occurring viruses that selectively replicate and kill cancer cells without harming normal tissues, may improve the situation by recruiting TILs, enhancing the anti-tumor immune response caused by tumor neoantigen presentation and turning cold tumors into hot [11].

In recent years, combinations of ICI with OV therapies have shown incredible anti-tumor benefits in large-scale preclinical studies [12–14]. In a previous study, the authors demonstrated that an oncolytic vaccinia virus (vvDD-CXCL11) markedly upregulated programmed cell death protein ligand-1 (PD-L1) in the TME, synergizing with anti-PD-L1 treatment to lead to over a 40% cure rate in models of aggressive peritoneal carcinomatosis from colon and ovarian cancers [15]. In another study, the authors reported the success of a phase 1b clinical trial testing the impact of Talimogene Laherparepvec (T-Vec) on cytotoxic T-cell infiltration and the therapeutic efficacy of pembrolizumab. Twenty-one patients with advanced melanoma were treated with T-Vec, followed by combination therapy with pembrolizumab. The confirmed objective response rate (ORR) was 62%, with a complete response rate of 33% per immune-related response criteria [16]. The above two studies demonstrated that OVs are more effective when combined with ICIs, typical of successful combinational immunotherapies.

Despite the success of ICI treatments for other tumor types, their clinical efficacy in HNSCC is less encouraging. Consequently, further clinical and translational studies are needed to understand the mechanisms better underpinning OVs and their potential synergism with ICIs, ultimately benefiting patients with HNSCC.

## Overview of OVs

### History of OVs

The history of fighting tumors with viruses can be traced back to the beginning of the last century. OVs were first reported in 1904 when a 42-year-old woman with leukemia contracted the influenza virus, and her tumor subsided. Then, in 1912, Pace announced that a dog bit a patient with cervical cancer, and after injection of the attenuated rabies virus vaccine, the patient’s cervical cancer was miraculously controlled [17]. The concept of oncolytic virotherapy was thus born, and related research quickly entered a period of fervor. In the 1920s, animal experiments proved that viruses could specifically infect and lyse the tumor cells of experimental mice [18]. Between 1950 and 1980, many clinical trials were performed in which patients with cancer were treated with wild-type or naturally attenuated viruses, including rabies virus, Newcastle disease virus, herpes simplex virus (HSV), and adenovirus(ADV). However, virotherapy research was slow to develop over the following decades, and virotherapy methods lacked efficacy and safety [19].

In 1991, Maritza et al. carried out thymidine kinase (TK) gene-knockout modification of HSV type 1 (HSV-1) for the first time. They demonstrated that dlsptk (a genetically engineered HSV-1 mutant) could destroy human glioblastoma cells in culture conditions or nude mice, which renewed the research community’s interest in viral anti-tumor therapy [20]. In 1995, Mineta et al. created a multi-gene mutant of HSV-1 (G207) with deletions of ICP34.5 and a lacZ gene insertion into the ICP6 gene [21] In the following years, Hunter [22] and Sundaresan [23] successively proved the efficacy of G207 in the treatment of malignant glioma in nude mice and the safety of G207 inoculation into the brains of mice and susceptible primates (New World owl monkeys). A higher intracranial dosage and better therapeutic effect were then obtained [24], and G207 represented the beginning of the search for a perfect combination of OV and gene recombination technology.

Since 1990, other genetically engineered oncolytic viruses and HSV have rapidly developed. Thus far, five genetically engineered oncolytic viruses have been approved for marketing as drugs (Table 1). In 2003, the recombinant human p53 adenovirus anti-tumor injection (Gendicine) was approved by the CFDA (China food and Drug Administration) to become the first gene therapy cancer drug released on the global market [25, 26]. In 2004, RIGVIR, the enteric cytopathic human orphan virus (ECHO-7), was approved in Latvia as an agent to treat melanoma [27]. Oncorine (H101) was approved in China for treating nasopharyngeal carcinoma in combination with 5-fluorouracil and cisplatin (chemotherapies in 2005). Oncorine is based on Ad vector serotype 5, in which the viral E1B-55k gene and four regions of the E3 gene guarantee its safe replication in p53-deficient tumor cells [28]. At present, the clinical efficacies of these products are not internationally recognized.

**Table 1:** Overview of global approval of oncolytic virus

Virus Name	Virus and genetic modification process	Indication	Approval authority	Listing date	Refs.
Gendicine	Recombinant adenovirus-p53	Advanced nasopharyngeal carcinoma and other solid tumors	CFDA (Changed to NMPA in 2018)	2003	[25, 26]
RIGVIR	ECHO-7 enterovirus	Melanoma and other solid tumors	Latvia	2004	[27]
Oncorine	Recombinant human adenovirus type 5, deleted E1B-55KD gene fragments	Nasopharyngeal carcinoma	CFDA (Changed to NMPA in 2018)	2005	[28]
Talimogene Laherparepvec (T-Vec)	HSV-1 ICP34.5 and ICP47 genes were knocked out, and GM-CSF was inserted at the ICP34.5 site	Advanced melanoma	FDA	2015	[32]
			Europe and Australia	2016	[33]
Delytact (Teserpaturev/G47∆)	HSV-1 created in the ICP34.5 and ICP47 genes. Further insertion of the Escherichia coli LacZ gene inactivates the ICP6 gene	Malignant glioma	Japan	2021	[34]

A further modified viral agent, G47Δ, is a triple-mutated third-generation oncolytic HSV-1 created by adding another deletion mutation to the genome of G207. To create G47Δ, two modifications were made to the ICP34.5 and ICP47 genes, followed by further insertion of the Escherichia coli LacZ gene, which inactivated the ICP6 gene. In 2013, clinical trials into the use of G47Δ for glioblastoma were conducted in Japan, and the agent was shown to kill cancer stem cells derived from human glioblastoma with efficiency [29–31]. T-Vec is a double-mutated HSV-1 that lacks the r34.5 and ȃ47 genes and inserted the human granulocyte–macrophage colony-stimulating factor (GM-CSF) gene into the deleted r34.5 loci. T-Vec was approved by the FDA for melanoma treatment in October 2015 and subsequently approved in Europe and Australia in 2016 [32, 33]. This approval marked the maturity of oncolytic virus technology and the formal recognition of the potential of oncolytic viruses as cancer treatments. In June 2021, the Japan Ministry of Health, Labor and Welfare (MHLW) approved, with conditions and time limits, Teserpaturev (Delytact/ G47∆) as a treatment for malignant glioma, and Delytact was the first oncolytic virus product approved globally for the treatment of malignant glioma [34].

### Anti-neoplastic mechanisms of OVs

#### Direct oncolysis

After an OV specifically infects neoplastic cells, it uses their energy, proliferates in tumor cells, and directly lyses them. OVs persist in cells and give rise to numerous offspring viruses that infect adjacent tumor cells until the body’s immune system clears the infection [35]. Some viral proteins, such as the E3-11.6kD protein and E4ORF4 protein expressed by adenovirus (ADV), also have direct cytotoxic effects on malignant cells [36, 37].

#### Induce systemic anti-tumor immunity

(1) Adaptive immunity activation: neoplastic cells release threat-associated molecular pattern molecules, pathogen-associated molecular pattern molecules, and a multiplicity of tumor-associated antigens (TAAs) after lysis to recruit immune cells, which infiltrate the tumor and activate CD4^+^, CD8^+^ T cells to induce antigen-specific T cell killing [38, 39]. (2) Tumor-suppressive microenvironment reversal: the TME is the soil for tumor survival and contains a plethora of immunosuppressive cells, such as immunomodulatory T cells (Tregs), and myelogenous suppressor cells (MDSCs), immunosuppressive cytokines, and molecules such as IL-10 and PD-L1. The inhibitory TME can protect the tumor from immune surveillance and promote vigorous tumor growth. The oncolytic storm promoted by an OV in the tumor can activate immune cells, such as dendritic cells (DCs) and natural killer cells, to enter the inhibitory state and promote the transformation of cold inhibitory tumors into hot tumors [40].

#### Destruction of the neoplastic vascular system

**Fig. 1:**
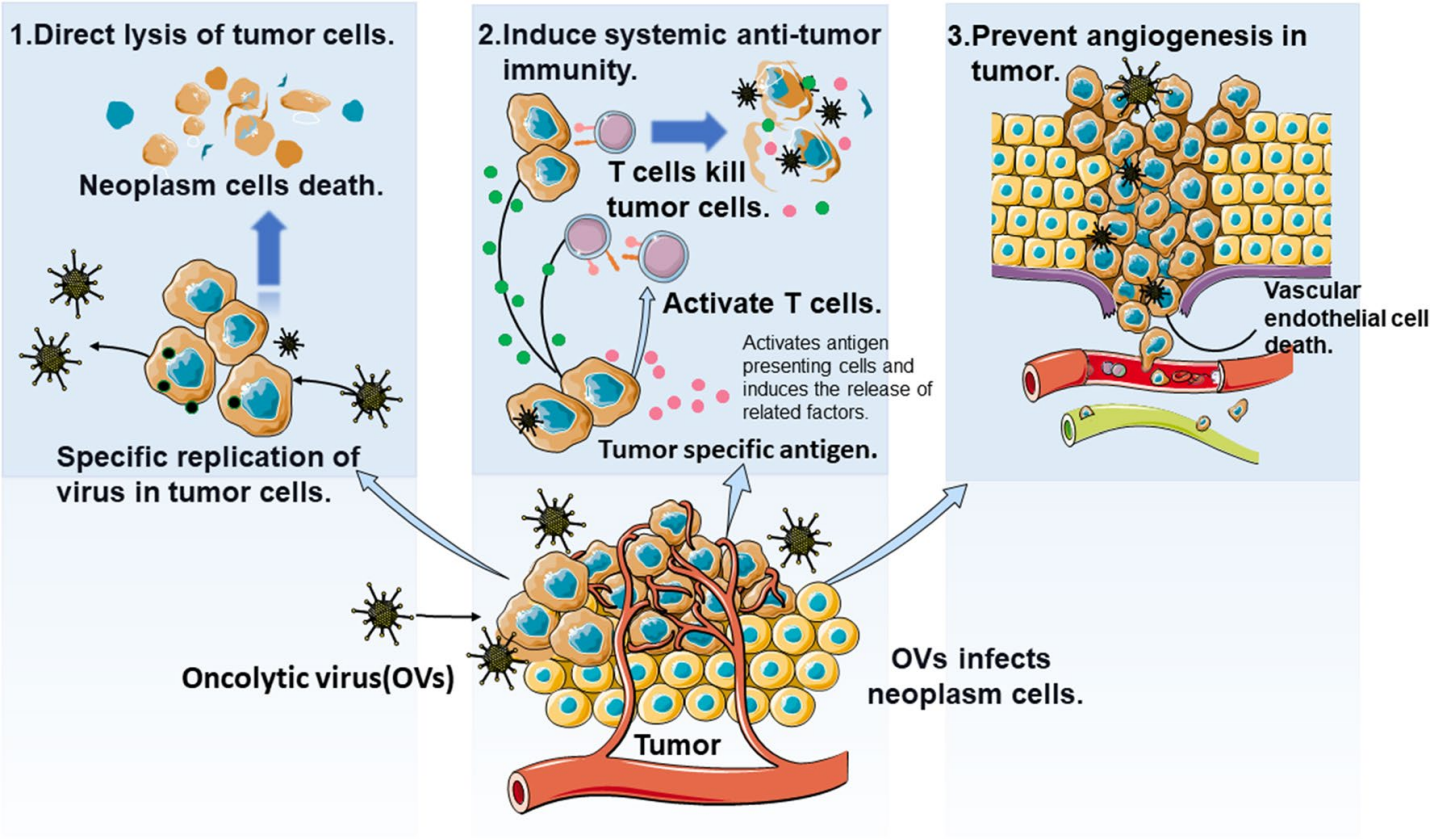
Anti-neoplastic mechanisms of OVs. (1) Direct oncolysis. (2) Induce systemic anti-tumor immunity. (3) Destruction of the neoplastic vascular system

The growth and metastasis of substantial tumors depend on a vascular system to provide nutrients to the tumor cells. Therefore, destroying tumor-related vascular systems can effectively inhibit tumor growth (Fig. 1).

### Oncolytic virus for treating HNSCC

#### Reoviruses

Reoviruses are wild-type double-stranded RNA viruses of the family Reoviridae [40]. In the 1960s, the first reovirus was isolated from humans’ and animals’ respiratory tracts and intestines. Because its pathogenic principle was not clear at that time, it was called respiratory tract (R), intestinal tract (E), and orphan (O) virus, i.e., reovirus for short. In the 1970s, Hashiro et al. found that wild-type reovirus could replicate in mammalian tumors and transformed cells but not in untransformed normal cells. This discovery prompted scientists to recognize the potential oncolytic ability of reoviruses [41].

In preclinical findings, all cell lines of common tumor types (over 80%) were susceptible to reoviral oncolysis [42–44], and intratumoral injection of reovirus caused tumor regression. It was reported that human glioma xenografts regressed by 80% after a single intratumoral injection of reovirus [44]. Similar efficacy was shown in breast [42], lymphoma [45], colon, and ovarian cancer [43] xenograft models.

Although reovirus has shown impressive anti-tumor effects in tumor models, its future potential as a mainstream cancer therapeutic is probably combined with other treatment modalities. The combination of reovirus and radiotherapy has enhanced cytotoxicity in various tumor cells in vitro and in vivo [46]. Using intratumoral reovirus and palliative radiotherapy to treat 23 patients with advanced solid tumors in a phase I dose-escalation study demonstrated the safety of combination therapy. It established that concurrent therapy administration of high-dose radiation does not diminish reovirus oncolysis [47]. In phase I/II study, an advanced 31 patients, including 14 with HNSCC, were treated with Reovirus type 3 Dearing (RT3D) in combination with taxanes. One (3.8%) patient had a complete response (CR), and 6 (23.1%) had a partial response (PR), suggesting that reovirus is also active in HNSCC [48]. The clinical trial of Reolysin in combination with paclitaxel and carboplatin in patients with head and neck cancer (NCT01166542, NCT00753038) has ended, and the results are not yet available.

Although wild attenuated viral strains have lower toxicity and better tumor-killing effects than other viruses, their clinical application has been unsatisfactory. In 1991, Martuza et al. carried out TK-knock-out gene transformation on HSV-1 for the first time, introducing the potential of creating transgenic OVs [20], Common genetically engineered OV, such as HSV, ADV, and VV.

#### Herpes simplex virus

HSV, the first oncolytic virus modified by gene mutation, is a double-stranded DNA virus with a large genome (152 kb) with ample transformation space that can accommodate the insertion of large fragments without suffering insertion mutations, making it an ideal oncolytic virus [40]. In addition to the above advantages, HSV has a vast host-species range, the ability to infect a variety of tumor cells, high infection efficiency, vigorous oncolytic activity, short oncolytic time, and the ability to kill tumor cells with a relatively low multiplicity of infection. Notably, antiviral drugs such as acyclovir and ganciclovir can effectively inhibit the proliferation of HSV; therefore, if necessary, treatment can be interrupted to ensure the patient’s safety. Presently, T-Vec, G207, and other HSV strains are being studied in clinical trials. For example, seventeen untreated individuals with Stage III/IV head and neck cancer were treated with T-Vec in a phase I/II study along with radiation and cisplatin. Patients received chemoradiotherapy (70 Gy in 35 fractions with concomitant cisplatin 100 mg/m^2^ on days 1,22, and 43) and dose-escalating T-Vec by an intratumoral injection on days 1, 22, 43, and 64. After finishing radiotherapy and chemotherapy, the patient had neck dissection six to eight weeks later. All patients have established local control with a relapse-free rate of 76.5% [49].

#### Adenoviruses

ADVs are capsid-free double-stranded DNA viruses with large genomes (about 35 kb) that facilitate the insertion of an extended gene fragment (10 kb) during genetic transformation. Various members of the ADV group infect humans and animals. Newborns and immunodeficient people are prone to disease, but normal cells can clear the infection quickly; therefore, ADVs have certain advantages for clinical practice application. Thus far, clinical trials have provided robust evidence that ADV therapy is relatively safe and has few side effects. In 1996, ONYX Pharmaceuticals developed and released ONYX-015, which is recognized as the most effective and promising oncolytic adenovirus. Fourteen patients with HNC who had failed to respond to conventional treatments were given a 5-day course of ONYX-015 intratumoral injections (4 × 10^9^–5 × 10^10^) PFU/d. The tumors of six individuals were significantly decreased, and several cases improved without any noticeable side effects. Forty head and neck cancer patients were enrolled in phase II clinical trial, and after receiving the ONYX-015 injection, the average tumor size was reduced by more than 50% [50]. In a clinical trial of ONYX-015 combined with standard treatment of 30 recurrent HNSCC cases, tumors shrank by more than 50% in 19 patients, accounting for 63% of all topics. In contrast, eight patients (27%) exhibited a complete response, and 11 (36%) had a partial response [51].

#### Vaccinia virus

VV, which belongs to the genus Orthopoxvirus, is the prototypical poxvirus and is well known for its use as a live-attenuated vaccine in the global eradication of smallpox. JX-594 is a genetically engineered vaccinia virus with a mutation in the *TK* gene, with the *GM-CSF* gene and *lac-Z* transgenes. JX-594 showed a good safety profile and induced repeatable tumor necrosis in various solid cancer types in clinical studies [52–55]. A phase I, open-label, dose-escalation trial of JX-594 in patients with advanced/metastatic solid tumors who failed to respond to standard therapy (NCT00625456), including 23 patients with malignant melanoma, non-small cell lung cancer, renal cell carcinoma, and HNSCC, has been completed, though results are not yet available. Although the application of VV in HNSCC has not been successful so far, further research is worth pursuing.

## Overview of ICIs

### Discovery of major immune checkpoints

**Fig. 2:**
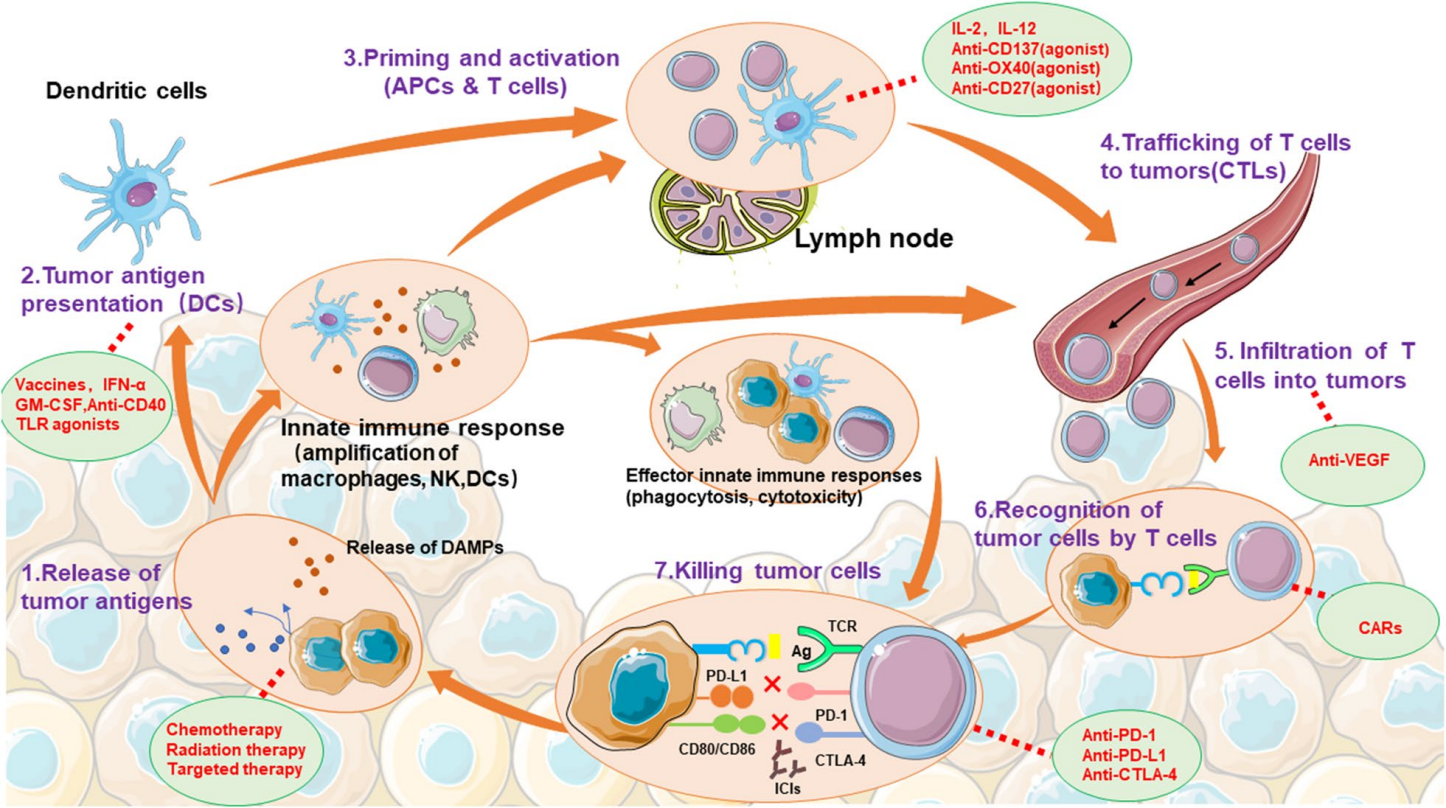
The mechanism of the immune system killing tumor cells and the mechanism of ICIs. The immune system of humans has an immune surveillance function. When tumors occur and develop, the body's immune system will kill the tumor cells as shown in the figure below. However, proteins on the surface of immune cells that prevent the over-activation of the immune system, i.e., immune checkpoints, are overexpressed and thus continuously send inhibitory signals to the immune system, resulting in tumor immune escape. For example, CTLA-4, PD-1 and PD-L1 are representative immune checkpoints. Thus, blocking the signal pathway of immune checkpoint interaction may be a powerful tool for tumor treatment

The immune system of humans has an immune surveillance function that can identify and precisely remove ‘non-self’ cells that have turned malignant to inhibit the occurrence and development of tumors. Chen and Mellman [56] put forward the concept of the cancer immune cycle in 2013, revealing the mechanism of the above immune system killing the tumor cells, which is divided into the following seven steps: (1) Release of tumor antigens. (2) Tumor antigen presentation(DCs). (3) Priming and activation (APCs & T cells). (4) Trafficking of T cells to tumors (CTLs). (5) Infiltration of T cells into tumors. (6) Recognition of tumor cells by T cells. (7) Clear tumor cells. However, when tumors occur and develop, proteins on the surface of immune cells that prevent the over-activation of the immune system, i.e., immune checkpoints, are overexpressed and thus continuously send inhibitory signals to the immune system, resulting in tumor immune escape. For example, cytotoxic T lymphocyte-associated protein-4 (CTLA-4), programmed cell death protein-1 (PD-1), and its programmed cell death protein ligand-1 (PD-L1) are representative immune checkpoints (Fig. 2).

### CTLA-4

In 1987, when screening mouse cytotoxic-T-cell-derived cDNA libraries, Brunet et al. came across a cDNA clone defining a sequence, *CTLA-4*, which could encode a 223-amino-acid protein belonging to the immunoglobulin superfamily. It consists of one V-like domain flanked by two hydrophobic regions, one of which has a structure suggestive of membrane anchoring. *CTLA-4* is mainly expressed in activated lymphocytes and is conducted with T-cell-mediated cytotoxicity in inducible models of this process. The mouse *CTLA-4* gene maps to band c of chromosome 1 [57]. In 1988, Lefranc et al. cloned the human *CTLA-4* gene isolated from a genomic library and designated *Hu-CTLA-4*; the *Hu-CTLA-4* gene exists as a single copy per human haploid genome and maps to band q33 of chromosome 2. They declared that the homology of the human and murine *CTLA-4* proteins is 76% [58].

In 1994, Bluestone et al. prepared an anti-CTLA-4 antibody to detect the effect of the activation of the *CTLA-4* receptor on mouse T cells and found that *CTLA-4* played a negative regulatory role in T cell activation [59]. Subsequently, in 1995, Bluestone et al. constructed CTLA-4-deficient mice to study the role of *CTLA-4* in vivo. *CTLA-4* deletion led to lymphocyte proliferation and catastrophic multi-organ tissue destruction, confirming CTLA-4’s negative regulatory role in T cell activation [60]. In the same year, the respective teams of Waterhouse and Allison confirmed this observation [61, 62]. In 1996, Allison et al. published a crucial scientific paper speculating that *CTLA-4* blockade removes the inhibitory signal from the costimulatory pathway and enhances the rejection of tumor cells. The authors injected BALB/c mice with B7-1-transfected 51BLimlO tumor cells and divided them into two groups based on the above hypothesis. They intraperitoneally injected anti-CTLA-4 or anti-CD28, respectively, and found that, compared with the anti-CD28-treated or untreated control mice, anti-CTLA-4 treated mice showed inhibited B7-51BLimlO tumor growth. This study first proposed the concept of the ICI and provided experimental animal evidence that the CTLA-4 antibody can enhance the anti-tumor immune response [63].

#### PD-1/PD-L1

PD-1 was the second immune checkpoint molecule targeted in tumor immunotherapy research. In 1992, the Tasuku Honjo team discovered and cloned *PD-1* from the apoptotic mouse B cell line and named it for its relationship to programmed cell death [64]. However, the direct relationship between *PD-1 a*nd programmed cell death was not confirmed in immediately subsequent studies. In 1999, Honjo et al. found that *PD-1-knock-out* mice developed some autoimmune diseases. In addition, *PD-1* was shown to be located on the surface of immune cells, such as activated T cells, B cells, and DCs, and a harmful immune-regulatory molecule like *CTLA-4* [65]. In the same year, Chen Lieping’s team described the protein B7-H1, the third member of the B7 family, which was proposed to play a negative regulatory role in the immune system [66]. In 2000, Freeman et al. also found a new molecule, B7, that can bind to PD-1 to inhibit the proliferation of T cells and the production of cytokines. As B7 is the ligand of *PD-1*, it was named *PD-L1* [67]. B7-H1 and PD-L1 were later shown to have similar structures but were identified at separate dates. With the growing reputation of *PD-1*, the name *PD-L1* has become more accepted by the scientific community. In 2002, Chen Lieping’s team confirmed that *PD-L1* is mainly expressed on the surface of immune cells, such as antigen-presenting cells, B cells, and T cells, which participate in tumor-related immune responses. However, its expression level is deficient in normal human tissues and high in lung, colorectal, ovarian, and other cancers [68]. In the following years, Chen Lieping’s and Honj’s teams found that blocking *PD-L1* significantly inhibited the growth of tumor cells in mice, and the development of tumors was wholly inhibited in PD-1-deficient mice. These results suggested that the *PD-1/PD-L1* pathway plays an essential role in tumor immunotherapy, and blocking this pathway may be a powerful tool for tumor treatment [68–71].

### Mechanism of ICIs

Under normal circumstances, to maintain the body’s immune homeostasis and avoid autoimmune disease and over-immunization and, thus, tissue damage, T cells need to be resistant and tolerant to autoantigens. Immune checkpoints play an essential role in the negative regulatory mechanism of T cells.

#### CTLA-4/CD28

CTLA-4 is a classical molecule involved in the negative-feedback regulation of T-cell responses and is expressed on the surface of activated T-cells. It is highly homologous to CD28 (the main co-stimulatory factor on the surface of T-cells) and binds to both CD80 (B7-1) and CD86 (B7-2) on the surface of antigen-presenting cells. CTLA-4 is responsible for delivering inhibitory signals to T cells so that they do not kill other cells, including tumor cells. Compared to CD28, CTLA-4 has a much higher affinity for CD80 and CD86 and, therefore, competes for and blocks the activating effect of CD28. In resting naïve T cells, CTLA-4 is mainly located intracellularly. When an antigen activates T cells, the immunosuppressive feedback system is activated. Stimulatory signals generated by signal-binding to the T cell receptor and CD28/B7 induce vesicles to carry CTLA-4 to the cell surface via cytokinesis. CTLA-4 competes with B7 for binding to generate negative regulatory signals, inhibiting full T cell activation and leading to tumor immune escape.

#### PD-1/PD-L1

PD-1 and PD-L1 are damaging regulatory molecules. PD-1 is expressed on the surface of activated T cells, while PD-L1 is widely found on many malignant cells, including HNSCC and antigen-presenting cells. Activated T lymphocytes can detect infected or mutated cells and release toxins to eliminate infected or abnormal cells. The PD-1/PD-L1 negative feedback immunosuppression system is activated when antigens activate T cells. The successful docking of PD-1 on the surface of T cells to PD-L1 on the surface of APCs leads to the apoptosis of activated lymphocytes—an immune mechanism that maintains the body’s homeostasis. Studies have shown that when PD-L1 is expressed on the surface of tumor cells, it can induce the apoptosis of tumor-specific T cells through the PD-1/PD-L1 signaling pathway, which leads to immune escape and tumor development over time. Blocking the binding of checkpoints to their ligands breaks the immune tolerance system and enhances the activity of immune cells. This can effectively promote the immune clearance of tumor cells and is also the mechanism of action of ICIs.

### Progress of research into ICIs

Thus far, one CTLA-4 inhibitor and several PD-1/PD-L1 inhibitors (Table 2) have been approved by the FDA for the clinical treatment of melanoma, lung cancer, HNSCC (Table 3), and other solid tumors.

**Table 2:** Currently approved immune checkpoint inhibitors

Drug name	Trade name	Target	Manufacturer	Age	Indication	References
Ipilimumab	Yervoy®	CTLA-4	Bristol-Meyers Squibb	2011	Melanoma, RCC	[77]
Nivolumab	Opdivo®	PD-1	Bristol-Meyers Squibb	2015	Melanoma, RCC, NSCLC, HNSCC, HL, Urothelial carcinoma, HCC, Colorectal cancer	[81]
Pembrolizumab	Keytruda®	PD-1	Merck	2014	Melanoma, NSCLC, HNSCC, PMBCL HL, Urothelial carcinoma, HCC, Cervical cancer, Gastric cancer, MSI-H/dMMR Solid Tumors	[82]
Cemiplimab-rwlc	Libtayo®	PD-1	Sanofi/ Regeneron	2018	CSCC, BCC, NSCLC	[88]
Atezolizumab	Tecentriq™	PD-L1	Genentech	2016	Urothelial carcinoma, NSCLC	[90]
Durvalumab	Imfinzi™	PD-L1	Astra Zeneca	2017	Urothelial carcinoma, NSCLC	[92]
Avelumab	Bavencio®	PD-L1	Merck/Pfizer	2017	Merkel cell carcinoma, RCC, Urothelial carcinoma	[97]

*RCC* renal cell cancer, *NSCLC* non-small cell lung cancer, *HNSCC* head and neck squamous cell carcinoma, *HCC* hepatocellular carcinoma, *HL* Hodgkin lymphoma(classic), *PMBCL* primary mediastinal B cell lymphoma, *MSI-H* microsatellite instability-high, *dMMR* mismatch repair gene-deficient, *CSCC* Cutaneous Squamous Cell Carcinoma, *BCC* Basal Cell Carcinoma

**Table 3:** Clinical trials using immune checkpoint inhibitors in HNSCC (May 2022 clinicaltrials.org)

Target	Drug name	Indication	Combined drugs	Enrollment	Phase	Status	Clinical Trials Gov Identifier/ references
CTLA-4	Ipilimumab	Advanced HNSCC	Cetuximab, Radiation	19	Ib	Active, not recruiting	[78]
PD-1	Nivolumab	HNSCC	Cetuximab, Methotrexate, Docetaxel	361	III	Completed	[81]
	Pembrolizumab	R/M HNSCC	/	192	Ib	Completed	[83–85]
		HNSCC	/	172	II	Completed	[86]
		R/M HNSCC	Cisplatin, Carboplatin, 5-FU, Cetuximab	882	III	Active, not recruiting	[87]
	Cemiplimab	Advanced Cancer, Advanced Malignancies	Hypo fractionated radiotherapy, Cyclophosphamide, Docetaxel, Carboplatin, GM-CSF, Paclitaxel, Pemetrexed	398	I	Completed	[89]
		R/M HNSCC, p16-Positive OSCC	Carboplatin, Paclitaxel	33	II	Recruiting	NCT04862650
		HNSCC	REGN6569	85	I	Recruiting	NCT04465487
		OSCC	ISA101B	86	II	Recruiting	NCT04398524
		HNSCC	/	44	II	Not yet recruiting	NCT04831450
PD-L1	Atezolizumab	R/M HNSCC	/	32	I	Completed	[91]
	Durvalumab	HNSCC	/	62	I/ II	Completed	[93]
		PD-L1-positive or -Negative R/M HNSCC	Tremelimumab, SoC (cetuximab, a taxanes, methotrexate, or a fluoropyrimidine)	736	III	Completed	[94]
		Locally Advanced HNSCC	Cetuximab, Radiation	69	I/ II	Not yet recruiting	[95]
		HNSCC, M HNSCC	Radiation, Tremelimumab	45	I/ II	Active, not recruiting	[96]
	Avelumab	Locally Advanced HNSCC	Cetuximab, Cisplatin, IMRT	707	III	Active, not recruiting	[98]

#### CTLA-4 inhibitors

##### Ipilimumab

Ipilimumab (Yervoy®, MDX010), an anti-CTLA-4 monoclonal antibody, was developed by the Medarex company in 1999 and has achieved remarkable results in numerous clinical studies into the treatment of patients with advanced melanoma [72–76]. In 2011, the FDA authorized MDX010 for advanced melanoma treatment [77]. This was the first ICI to receive FDA approval for commercial use, ushering in a new era of tumor immunotherapy. A phase I clinical trial (NCT01935921) of cetuximab, radiotherapy, and ipilimumab to treat locally advanced head and neck cancer showed that the RP2D for ipilimumab plus standard cetuximab-radiotherapy is 1 mg/kg [78]. The regimen is tolerable and yields acceptable survival without cytotoxic chemotherapy. Tremelimumab is another anti-CTLA-4 antibody effective for melanoma, gastric cancer, esophageal cancer, and non-small cell lung cancer. In HNSCC patients, tremelimumab is mainly combined with durvalumab. Unfortunately, both CONDOR [79] and EAGLE [80] studies show that the addition of tremelimumab cannot improve the ORR and OS of patients. We look forward to the surprise of the ongoing clinical study of the durvalumab + tremelimumab combination regimen.

#### PD-1 inhibitors

##### Nivolumab

Nivolumab (Opdivo®) is the world’s first monoclonal antibody to be approved that targets PD-1, and it has significant therapeutic effects in patients with recurrence or metastasis (R/M) of HNSCC. In a randomized, open-label, phase III clinical trial, 361 patients with R/M HNSCC who relapsed within six months after platinum chemotherapy were randomly assigned nivolumab (N = 240) or conventional treatment (e.g., methotrexate, docetaxel, or cetuximab) (N = 121). The median OS in the nivolumab group was 7.5 months (95% confidence interval [CI], 5.5–9.1), while the median OS in the standard treatment group was 5.1 months (95% CI, 4.0–6.0). The nivolumab group had a 6-month progression-free survival (PFS) rate of 19.7%, while the conventional treatment group had a rate of 9.9%. The nivolumab group had a 13.3% effective rate, while the standard treatment group had a 5.8% effective rate. In the nivolumab group, 13.1% of patients experienced grade 3 or 4 treatment-related side events compared to 35.1% in the usual therapy group. OS was significantly longer in the nivolumab group than in the standard treatment group (hazard ratio of death, 0.70, 97.73% CI, 0.51 to 0.96, P = 0.01). Because of the test results of Checkmate-141, the FDA approved nivolumab in November 2016 for treating R/M HNSCC patients who failed to respond to platinum drugs [81]. In 2020, Even et al. reported the research results of phase II clinical study TOPNIVO (NCT03226756) with a follow-up of 23.5 months at the ESMO conference: 268 of the 343 R/M HNSCC patients who received nivolumab died. The median OS of the population was 7.5 months (95% CI: 6.5–8.9), the median PFS was 1.8 months (95% CI: 1.8–1.9), and the ORR was 56.0%. The study results are similar to Checkmate-141, which reflects the stability of nivolumab in treating HNSCC.

##### Pembrolizumab

Pembrolizumab (Keytruda®), approved by FDA for the first time to treat malignant melanoma in 2014 [82], is another anti-PD-1 antibody that can be used to treat patients with R/M HNSCC. Sixty patients with recurrent or metastatic HNSCC were treated with pembrolizumab (10 mg/kg, once every two weeks) in a phase Ib clinical trial (KEYNOTE-012) until disease progression, unacceptable adverse events, or other disorders made treatment impossible. 18% of patients went into remission, with a PFS and OS of 2 and 13 months, respectively [83]. Then, 132 patients with R/M HNSCC were involved in an extensive KEYNOTE-012 investigation; six-month PFS and OS were 23% and 59%, respectively [84, 85]. A phase II clinical study to test the efficacy of pembrolizumab, dubbed KEYNOTE-055, involved 172 patients with recurrent or metastatic HNSCC for whom the disease had progressed within six months after therapy with platinum medication in combination with cetuximab. Every three weeks, the participants were given 200 mg of pembrolizumab. The remission rate was 16%, PFS was 2.1 months, and OS was 8.0 months [86]. A randomized phase 3 study (KEYNOTE-048) was conducted on patients with R/M HNSCC who could not be cured locally without treatment. From April 20, 2015, to January 17, 2017, 882 candidates were included. They were assigned to receive pembrolizumab alone (n = 301), pembrolizumab combined chemotherapy (n = 281), or cetuximab combined chemotherapy (n = 300). The results showed that in the subgroup with positive PD-L1, the median OS pembrolizumab was longer than that of the cetuximab combined chemotherapy group, regardless of whether it was combined with chemotherapy. The safety of the three groups was acceptable, but neither pembrolizumab alone nor combined chemotherapy could improve PFS. According to this, FDA approved that pembrolizumab combined with chemotherapy (cisplatin and 5-FU) can be used as the first-line treatment scheme for all R/M HNSCC patients, and pembrolizumab can be used as the first-line treatment scheme for R / M HNSCC patients with CPS ≥ 1 [87].

##### Cemiplimab-rwlc

Cemiplimab-rwlc (LIBTAYO®, Cemiplimab) is a monoclonal antibody that targets PD-L1. It has been approved by FDA to treat advanced skin squamous cell carcinoma [88]. The drug is under research and can be used to treat various cancers. A stage I clinical trial of Cemiplimab-rwlc, radiotherapy, cyclophosphamide, and GM-CSF in patients with R/M HNSCC (NCT02383212) revealed that the impact of Cemiplimab-rwlc in conjunction with radiotherapy, cyclophosphamide, and GM-CSF might be similar to anti-PD‐1 monotherapy [81]. One explanation for the failure of this combination to exceed single agent data may be the enrollment of patients who were heavily pretreated, with 100% of patients receiving platinum‐based chemotherapy, 60% monoclonal antibodies, 60% pyrimidine analog, 53% taxanes, and 93.3% prior radiotherapy. This can lead to a general immunosuppressive state, potentially abrogating the immunostimulatory effect of the regimen [89]. Currently, multiple clinical investigations into Cemiplimab-rwlc monotherapy in parallel with chemotherapeutic or targeted drugs for treating R/M HNSCC are in progress (NCT04862650, NCT04831450, NCT04465487, NCT04398524), and the outcomes are expected to be encouraging.

#### PD-L1 inhibitors

##### Atezolizumab

Atezolizumab (Tecentriq™) [90] is a PD-L1 monoclonal antibody made up of synthetic IgG1 molecules. In a phase, I clinical trial, 6% of the 32 individuals with HNC included in the survey experienced adverse effects. The median response time for 22% of the patients was 7.4 months (2.8–45.8 months). The median PFS time was 2.6 months (range 0.5–48.4 months), with a 6.0-month median OS time (range 0.5–51.6 months). There was no correlation between response and HPV status or PD-L1 expression level. To some degree, the trial demonstrated that atezolizumab is safe, tolerable, and has high clinical effectiveness; however, the sample size was shallow. In the future, the investigation should be broadened to compare atezolizumab with presently used treatment, both alone and when employed in conjunction with other therapies [91].

##### Durvalumab

Durvalumab (Imfinzi™, AstraZeneca) [92] is an essential PD-L1 inhibitor used in the treatment of R/M HNSCC. In a clinical trial completed last year, the incidence of drug-related adverse events in 62 patients with HNSCC was 59.7%. The incidence of grade 3–4 adverse events was 9.7%, the ORR was 6.5%, the medium OS was 8.4 months, the 6-month survival rate was 62%, and the 12-month OS rate was 38%, indicating that durvalumab is safe and effective for the treatment of HNSCC patients [93]. Zandberg et al. applied durvalumab to patients with R/M HNSCC with high expression of PD-L1 (expression rate greater or equal to 25%), injecting 10 mg/kg of the drug intravenously every two weeks. The primary endpoint was the ORR, and the secondary was PFS and OS. The median PFS and OS of the treated patients (n = 112) were 14.6% and 33.6% at 12 months, respectively. Therefore, durvalumab has acceptable safety and antitumor activity in patients with R/M HNSCC with high levels of PD-L1 [94]. To improve clinical efficacy, the PD-L1 antibody can be combined with other immunotherapies for HNSCC [95, 96]. CONDOR [79] study evaluated the effectiveness of durvalumab in R/M HNSCC patients with low PD-L1 expression (< 25% of tumor tissues express PD-L1) or no word. 267 patients received durvalumab, tremelimumab monotherapy, and durvalumab combined with tremelimumab, respectively. The results showed that the median OS of the three groups was 6.0 months, 5.5 months, and 7.6 months respectively; ORR was 9.2%, 1.6%, and 7.8%, respectively; The rates of TRAEs were 63.1%, 55.4%, and 57.9% respectively; The incidence of grade 3–4 TRAEs was 12.3%, 16.9%, and 15.8% respectively. The most common TRAEs were diarrhea and fatigue. Five iTRAEs were reported in the durvalumab monotherapy group, and eight iTRAEs in the durvalumab + tremelimumab combination group. One treatment-related death was reported in the study. The patient developed treatment-related grade 3 respiratory failure on day 38 after the first treatment cycle and died of disease progression on day 50. This is consistent with the conclusion of relevant studies on nivolumab that "AES in ICIs treatment mainly occurs in the early stage of medication.” It is worth noting that similar percentages of HPV-positive patients (28.1%, 25.6%, and 24.0%, respectively) appeared in CONDOR, Checkmate-141, and KEYNOTE-040 studies, but HPV-positive patients did not show higher efficacy in CONDOR studies. HAWK and CONDOR studies showed that in the second-line treatment scheme of R/M HNSCC, whether PD-L1 was expressed or not, durvalumab treatment was beneficial. Still, the addition of tremelimumab did not increase the benefit. EAGLE [80], a prospective phase III clinical trial, compared the efficacy of durvalumab, durvalumab combined with tremelimumab, and standard of care (SoC) in patients with R/M HNSCC. The results showed that the OS of the three groups was 7.6 months, 6.5 months, and 8.3 months respectively; The median PFS was 2.1, 2.0, and 3.7 months, respectively; ORR was 17.9%, 18.2%, and 17.3% respectively; TRAEs ≥ grade 3 were 10.1%, 16.3%, and 24.2% respectively. There were 9, 10, and 10 patients who stopped taking TRAEs, respectively; 4, 2, and 9 patients died due to TRAEs. Bleeding-related TRAEs are rare. In the durvalumab + tremelimumab group, the most common grade 3–4 TRAES were asthenia (5 cases), anemia (4 points), and fatigue (3 issues). The results show that compared with SoC, the incidence of TRAES ≥ grade 3 of durvalumab monotherapy and tremelimumab combined with tremelimumab is slightly lower. Still, it cannot improve the OS of patients. It is a pity that durvalumab cannot replace the first-line standard chemotherapy regimen for R / M HNSCC. The following clinical study of durvalumab combined with a traditional treatment regimen is expected to open new therapeutic directions.

##### Avelumab

In 2017, the FDA authorized avelumab (Baveneio®) as the first anti-PD-L1 (IgG1) monoclonal antibody for clinical use [97]. Relevant clinical studies indicated that avelumab has a therapeutic impact on HNSCC. A total of 153 patients with platinum-refractory R/M HNSCC were included in a phase I Javelin Solid Tumor trial to assess the efficacy and safety of avelumab, and 12 patients (7.8%) did not meet the platinum chemotherapy conditions. The objective remission rate set by the researchers was 13.1% (95% CI 8.2–19.5%). The investigators determined that the median PFS was 1.8 months (95% CI 1.4–2.7%), and the median OS was 8.0 months (95% CI 6.5–10.2%). TRAE occurred in 83 patients (54.2%), with ten patients experiencing grade 3 TRAE (6.5%). Fatigue (n = 19, 12.4%), fever (n = 14, 9.2%), pruritus (n = 12, 7.8%), and chills (n = 11, 7.2%) were the most prevalent TRAEs, with no treatment-related fatalities [98]. The use of avelumab to treat HNSCC is still in its early stages. More phase II and phase III clinical trials to explore the efficacy of avelumab are in progress.

## OVs in combination with ICIs for treating HNSCC

**Fig. 3:**
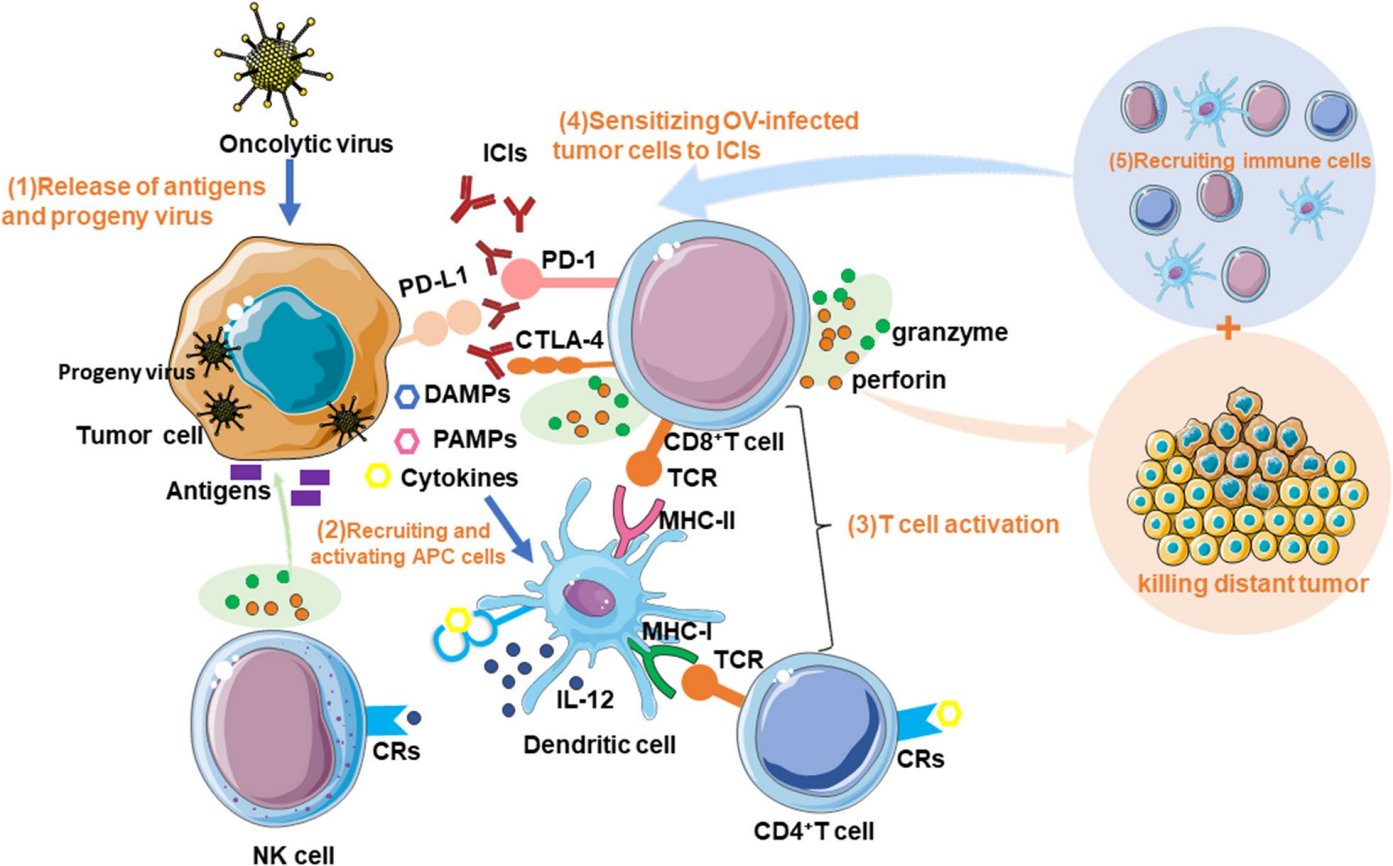
Mechanism of OVs combined with ICIs to stimulate anti-tumor immunity.** (**1) OVs enter tumor cells and undergo viral replication, leading to lysis and the release of danger-associated molecular pattern signals (DAMPs), pathogen-associated molecular patterns (PAMPs), tumor-associated antigens (TAAs), and pro-inflammatory cytokines. Viral progeny is also released, spreading to and infecting neighboring tumor cells. (2) These molecules recruit and activate antigen-presenting cells (APCs), such as dendritic cells (DCs), and promote the maturation of DCs through costimulatory markers while promoting the release of pro-inflammatory cytokines such as interleukin 12 (IL-12) from DCs and recognition by cytokine receptors (CRs) on T cells and NK cells. (3) Mature DCs cross-present antigens to CD4^+^ and CD8^+^ T cells via the major histocompatibility complex (MHC) and induce their expansion and activation. T cells and NK cells eventually lyse tumor cells by releasing perforin, granzyme, and cytokines. (4) OVs infection leads to increased expression of immune checkpoint molecules such as PD-L1 and CTLA-4, thereby increasing the expression of the therapeutic targets of ICIs and sensitizing OV-infected tumor cells to ICIs. (5) In addition, local injection of OVs into individual tumor sites induces a distant effect, causing T cells to migrate to the site of metastatic disease, recognizing and killing distant tumor cells. Cytokines and chemokines released in the tumor microenvironment can recruit immune cells for concerted anti-tumor activity

Tumor patients treated with ICIs have demonstrated apparent survival advantages in clinical trials. Nonetheless, increasing evidence suggests that researchers must overcome several fundamental constraints. To begin with, some patients have suffered severe immune-related side effects (iRAEs) after ICI use [99]. Secondly, ICI treatment only benefits a small percentage of cancer patients and is ineffective for immunologically cold tumors with low TIL levels [100]. Thirdly, according to several studies, most tumors’ TMEs, including HNSCC tumors, are highly immunosuppressive, making them immunologically cold. Therefore, converting a hard tumor microenvironment into a hot environment will aid ICI effectiveness. OVs have the unique ability to turn a cold TME into a hot one, increasing immune cell and lymphocyte infiltration (Fig. 3). Therefore, OVs combined with various cancer immunotherapy medicines have excellent treatment prospects and are ideal accompaniments to ICIs in combination therapies to overcome ICIs’ limitations in treating HNSCC (Table 3).

### Reovirus + PD-1/PD-L1 blockade

#### Preclinical experiments

Numerous preclinical studies have found that combining a PD-1 antibody and reovirus is more effective than each monotherapy. Rajani et al. found that reovirus-treated B16 melanoma mice showed delayed tumor growth but no significant survival improvements compared to B16 melanoma mice treated with phosphate-buffered saline. Systemic treatment with anti-PD-1 antibodies did not provide survival benefits. However, when the reovirus was combined with a PD-1 antibody, survival was significantly prolonged compared to reovirus monotherapy (P < 0.001), and the treatment cured approximately 40% of mice [101]. Studies have shown that a combination of reovirus and PD-L1 antibodies can also be beneficial against tumors. In vitro experiments by Mostafa et al. [102] and Kelly et al. [103] demonstrated that reoviruses upregulated PD-L1 expression in multiple myeloma (MM) cells. The oncolytic reovirus pelareorep (Reolysin) has significant immunomodulatory effects and has shown promising preclinical efficacy in MM models, and was also found to be safe and well-tolerated in early MM clinical trials. Kelly et al. constructed a multiple myeloma model to further investigate the in vivo therapeutic potential of Reolysin-based PD-L1 expression. They showed that the skeletal tumor load was almost completely regressed in mice treated with a combination of Reolysin and PD-L1 inhibitors. Ultimately, Reolysin and PD-L1 antibody therapy were shown to effectively treat MM syngeneic mouse models with many landmark features [103]. llett et al. [104] and Samson et al. [105] reported the benefits of similar combination regimens in mouse models of melanoma and glioma, respectively.

#### Clinical experiments

There is no clinical trial of Reovirus combined with ICI in treating HNSCC on the clinical trials gov website. A Phase 1b Study of Pembrolizumab (KEYTRUDA®) in Combination With REOLYSIN® (Pelareorep) and Chemotherapy in Patients with Advanced Pancreatic Adenocarcinoma (NCT02620423) reported that intravenous Reolysin combined with chemotherapy (5-fluorouracil, gemcitabine, or irinotecan) and pembrolizumab was a safe and effective strategy for treating advanced pancreatic cancer. Disease control was achieved in 3 of the ten patients evaluable for efficacy; one patient achieved partial remission within 17.4 months, and the other two had respective stable disease terms of 9 and 4 months. Treatment was well-tolerated, and adverse events were mainly graded 1 or 2 treatment-related, e.g., flu-like symptoms [106].

### HSV + anti-PD-1/PD-L1

#### Preclinical experiment

HSV-1 has been extensively studied regarding its role as the backbone of OV therapy. Several preclinical and early clinical trials have demonstrated its benefits when combined with ICIs. The rhabdomyosarcoma mice treated with anti-PD-1 and HSV1716 had longer survival times than untreated mice or mice receiving monotherapy. The PD-1 antibody alone did not significantly alter the overall T cell population in the tumors or spleens of mice. At the same time, intratumoral injection of HSV1716 promoted both a local and a systemic increase in CD44^+^ memory T cells (CD4^+^ and CD8^+^) [107]. ONCR-177 is a gene-engineered recombinant HSV equipped with five transgenes of IL-12, FLT3LG extracellular structural domain, CCL4, and antagonists of the immune checkpoints PD-1 and CTLA-4. In vivo assays demonstrated that the intratumoral administration of a mouse ONCR-177-replacement virus, mONCR-171, effectively treated a group of homozygous bilateral mouse tumor models( mouse colon carcinoma, B-cell lymphoma, and melanoma), leading to partial or complete tumor regression, translating to significantly enhanced survival and triggering a protective memory response. The addition of systemic anti-PD-1 also improved the efficacy of mONCR-171, particularly in distant tumors [108].

#### Clinical experiments

##### T-Vec

With the success of T-Vec monotherapy, including its ability to modulate the immune response to tumors, there has been speculation that it could lead to a more significant therapeutic benefit in combination with ICIs. A phase 1b multicenter clinical trial of T-Vec in combination with intravenous pembrolizumab for platinum-refractory R/M HNSCC (NCT02626000) enrolled 36 patients. The primary endpoint was the incidence of dose-limiting toxicity (DLT), and critical secondary endpoints included the objective remission rates, PFS per immune-related RECIST (irRECIST (Response Evaluation Criteria in Solid Tumors)), OS, and the safety of each irRECIST. At the time of evaluation, one patient experienced T-Vec-related lethal disease (arterial bleeding), and 20 (55.6%) and 21 (58.3%) patients experienced T-Vec and pembrolizumab-related adverse events, respectively. There were no treatment-related fatal adverse events except for DLT. The median PFS and OS were 3.0 months (95% Cl, 2.0–5.8) and 5.8 months (95% Cl, 2.9–11.4), respectively. Therefore, the combination of T-Vec and pembrolizumab demonstrated a tolerable safety profile as a therapy for R/M HNSCC, and the efficacy of the mixture was like that of pembrolizumab monotherapy in historical HNSCC studies [109].

##### ONCR-177

A clinical trial of ONCR-177 alone or combined with a PD-1 antibody to treat advanced and refractory cutaneous, subcutaneous, and metastatic lymph node solid tumors and HNSCC (NCT04348916) is currently under recruitment. ONCR-177 is an intratumorally administered oncolytic immunotherapy comprised of a genetically engineered HSV-1 that selectively replicates in tumor tissue. This first-in-human (FIH) Phase 1 dose escalation and expansion study will determine the maximum tolerated dose (MTD) and recommended phase 2 dose (RP2D) of ONCR-177 as a monotherapy and in combination with pembrolizumab in subjects with advanced and refractory cutaneous, subcutaneous, or metastatic nodal solid tumors, or with Liver Metastases of Solid Tumors, and Confirm the safety of ONCR-177 administration in combination with pembrolizumab.

### ADV + anti-PD-1/PD-L1/CTLA-4

#### Preclinical experiments

ADVs have been extensively studied, and their therapeutic roles, combined with ICIs, are the focus of active research. ISF35 is a nonreplicating adenovirus that encodes a human-mouse chimeric, an optimized variant of CD40L that functions as a CD40 agonist. In mouse melanomas, Singh et al. discovered that intratumoral injection of ISF35 increased tumor-specific CD8^+^ T cell numbers and PD-1 expression. ISF35, in combination with the PD-1 antibody, had much higher anti-tumor efficacy than ISF35 and the PD-1 antibody alone. On the other hand, a triple combination of ISF35, anti-PD-1, and anti-CTLA-4 resulted in complete tumor eradication. These findings imply that intratumoral CD40 activation with ISF35 combined with checkpoint inhibition is an effective treatment for multifocal malignancies. [110]. TILT-123 is an ADV with two potent anti-tumor cytokines (TNF-α and IL-2) [12]. TNF-α and interleukin-2 (IL-2) displayed pro-inflammatory effects and increased T cell trafficking, activation, and proliferation after lysing a mouse melanoma treated with TILT-123, according to Cevera-Carrascon et al. The modified adenovirus, combined with the PD-1 antibody, improved tumor growth control, and OS compared to either single treatment [12].

#### Clinical experiments

A study into the use of TILT-123 and avelumab for treating melanoma and HNSCC after anti-PD-L1 therapy (Aventil) (NCT05222932) has been designed but has not yet begun enrollment. A clinical trial is being conducted on the use of OBP-301(Telemelysin), a serotype-5 oAd (oncolytic adenovirus) that replicates under human telomerase reverse transcriptase promoter control [111], with pembrolizumab for advanced solid tumors (NCT03172819). The continued increase in clinical studies on ADV combined with anti-PD-1/PD-L1 reflects this strategy’s excitement.

### VV + anti-PD-1/PD-L1

#### Preclinical experiments

Preclinical trials have shown that VV has good antitumor efficacy and safety, and several research groups have investigated whether combining it with ICIs would lead to more significant therapeutic benefits. Liu et al. conjectured that VV would attract effector T cells and induce PD-L1 expression by cancer and immune cells in tumors. They tested their conjecture with mouse models of colon and ovarian cancer. VV combined with ICIs reduced the number of PD-L1^+^ cells and promoted the non-redundant tumor infiltration of effector CD8^+^CD4^+^ T cells while increasing the expression of IFN-γ, ICOS, granzyme B, and perforin. Furthermore, the combination therapy also reduced viral-induced PD-L1^+^ DCs, MDSCs, TAM, and Treg cell population, severely depleting the co-suppressed molecular-double-positive PD-1^+^CD8^+^ T cell population, resulting in reduced tumor load and improved survival [15].

Kowalsky et al. constructed vvDDIL15-Ra, a novel lysogenic poxvirus (VV) expressing an IL-15 superagonist (a fusion protein of IL-15 and IL-15R-α), which has similar replication efficiency to the parental virus vvDD. And they also found it to be more effective against tumors and effectively prolonged survival in mice with colon and ovarian cancers than the parental virus vvDD. Compared with anti-PD-1 or vvDD-IL-15R-α monotherapy, vvDDIL-15R-α combined with the PD-1 antibody induced significant tumor regression and prolonged the survival of colon or ovarian cancer mice [112]. Smith et al. found that isolated limb perfusion of VV followed by the PD-1 antibody for the treatment of soft tissue sarcoma, the viral ILP augmented the response to PD-1 blockade [113].

#### Clinical experiment

Although the JX-594 monotherapy has encouraging antitumor immunostimulatory properties for multiple solid cancer types, there have been few clinical trials into JX-594 in combination with ICIs against HNSCC, which warrants pursuit. Clinical investigations utilizing JX-594 in conjunction with ICIs against different malignancies are ongoing. For example, a clinical trial using recombinant VV and cemiplimab for renal cell carcinoma (NCT03294083) is now recruiting participants. A clinical trial of JX-594 intratumoral injection combined with ipilimumab in metastatic/advanced solid tumors (NCT02977156) is ongoing.

## Discussion

**Fig. 4:**
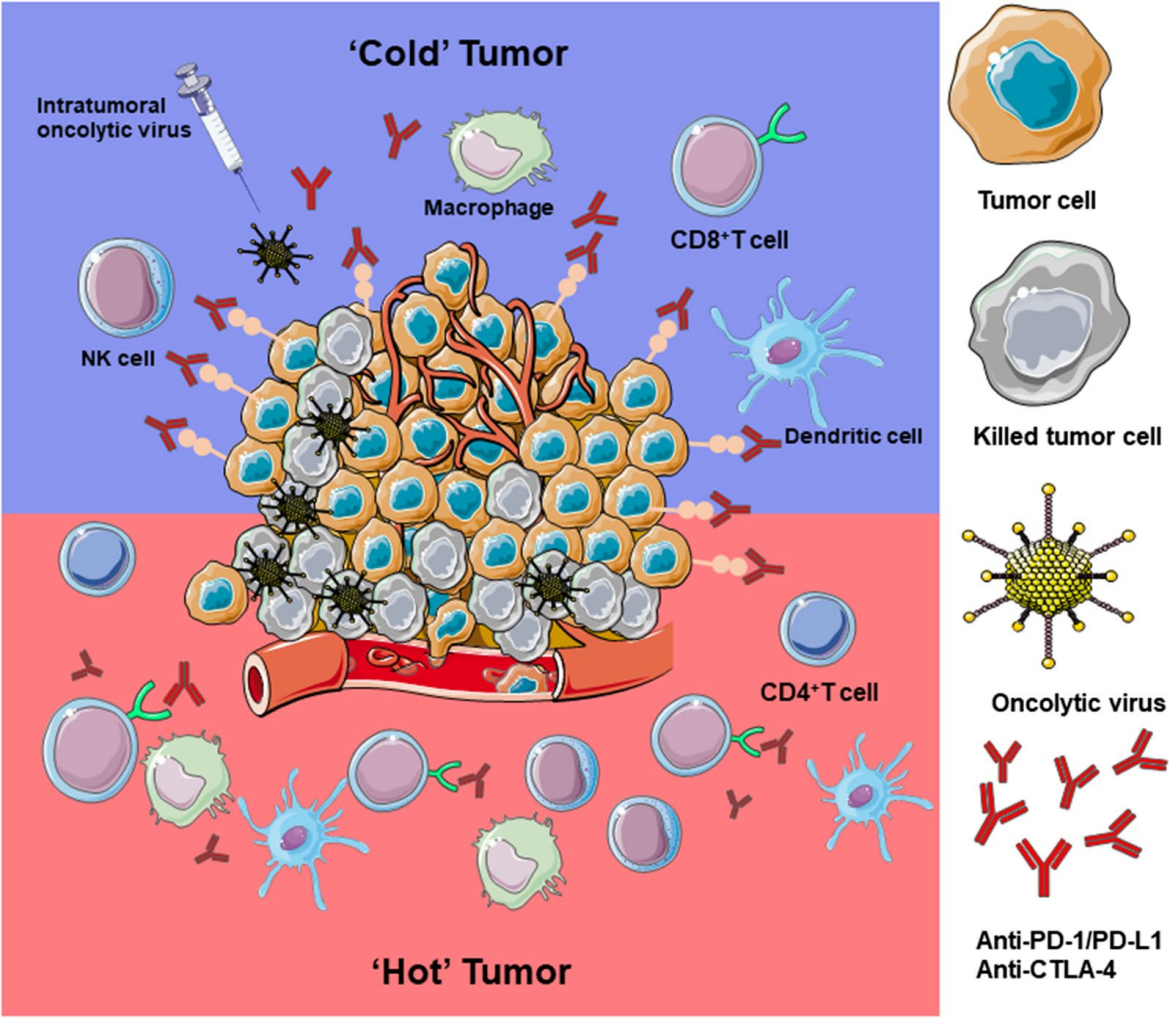
OVs combined with ICIs are complementary to each other. OVs infecting tumor cells attract many APCs to the tumor injection site, stimulating CD8^+^ T production and increasing the infiltration of CD8^+^ T cells in the tumor microenvironment, transforming a "cold" tumor into a "hot" tumor. The effect of OVs stimulates the expression of PD-L1 on the surface of tumor cells, and ICIs can block the binding of PD-L1 and PD-1, thus relieving the immunosuppressive effect. The ICIs can enhance the anti-tumor immune response triggered by OVs. Therefore, local injection of OVs to activate the systemic anti-tumor immune response increases the infiltration of CD8^+^ T cells and promotes the expression of PD-L1, followed by the administration of ICIs to enhance the therapeutic effect

A significant obstacle to the treatment of HNSCC patients is the high rate of recurrence and metastasis. Previous studies have shown that more than 60% of HNSCC cases are locally advanced or metastatic at the time of diagnosis. Once recurrence or metastasis occurs, the 5-year survival rate of HNSCC is less than 40% [114, 115]. In recent years, immune checkpoint inhibitors (ICIs) have become a promising strategy for treating tumors. However, the clinical effect on head and neck squamous cell carcinoma (HNSCC) is poor. The combination of oncolytic virus therapy can break through the limitations of ICIs in treating HNSCC and improve the sensitivity of head and neck squamous cells to ICIs. The application of OVs combined with ICIs against tumors has shown success in many preclinical studies and has started to become the focus of clinical trials. OVs combined with ICIs are effective against tumors and improve patient survival compared to monotherapies due to their complementary advantages. ICIs are effective against tumors with high levels of TILs, i.e., immunologically hot tumors. Furthermore, they enhance the anti-tumor immune response induced by tumor neoantigen presentation, transforming a cold tumor into a hot one. After OVs infect tumor cells, they promote the release of cytokines (e.g., GM-CSF, TNF-α, IFN) and TAA from tumor cells, attracting many APCs to the tumor injection site, which in turn stimulates the production of CD8^+^ T cells and increases their infiltration into the TME, preparing the ICIs for action. The release of inflammatory factors stimulates the expression of PD-L1 on the surface of tumor cells, and when it binds to PD-1 on the surface of T cells, an immunosuppressive effect occurs (Fig. 4). For example, a preclinical investigation verified that viral oncolysis dramatically increased PD-L1 expression in primary liver tumors and lung metastases, which completely inhibited the spread of tumor cells and eliminated resistance to PD-1 blocking therapy [116]. Therefore, ICIs and the immunological counter-regulator response to viral infection may work together.

OVs, which induce the lysis of tumor cells and change the local immune microenvironment, can help increase the function of immune effector cells at the tumor site and improve tumor cell sensitivity to ICIs, making combination therapies of OVs and ICIs desirable anti-tumor strategies. However, at present, the drug delivery method for oncolytic viruses is generally used for intratumoral injection. This method can effectively exclude the body's antiviral immune response. Still, it is difficult to inject intratumorally into tumors deep in the body directly, such as gliomas. It can only be used to treat detected solid tumors, which significantly limits the clinical use of OVs. Intravenous administration can solve this problem and inhibit both primary and metastatic lesions. Still, the greatest obstacle is overcoming the body's antiviral immune response to achieve effective therapy concentrations. In other words, it remains difficult to deliver OVs to all primary and metastatic tumor sites to achieve the desired impact. In distant metastatic lesions with modest T-cell infiltration, the therapeutic benefit of OVs combined with ICIs may be negligible [117]. So new vectors need to be found to deliver OVs to tumor tissues for therapeutic use.

Additionally, is the recombinant oncolytic virus loaded with the anti PD-1/ CTLA-4 antibody gene effective against cancer? Or is simultaneously administering immune checkpoint inhibitors and using oncolytic viruses to treat cancer the best option? What is the best time for antibody delivery in the latter, and how to determine the timing and dose of the combination? How to correctly select qualified patients and the disadvantages of the combination strategy. These issues still require additional verification by massive research. Most biomarkers, such as PD-L1 and CTLA-4 expression, can guide the precise use of ICIs. However, some patients will still benefit from them even if they do not have these biomarkers. Thus, finding new biomarkers that are more relevant and responsive to treatment and further evaluating and supporting them through future research is also an essential direction for future research.

## Data Availability

The results/data/figures in this manuscript have not been published elsewhere, nor are they under consideration by another publisher.
